# Exploring Sertoli Cells’ Innate Bulwark Role Against Infections: *In Vitro* Performances on *Candida tropicalis* Biofilms

**DOI:** 10.3390/cells14070495

**Published:** 2025-03-26

**Authors:** Iva Arato, Stefano Giovagnoli, Luca Roscini, Mario Calvitti, Catia Bellucci, Cinzia Lilli, Elena Eugeni, Stefano Brancorsini, Gianluigi Cardinali, Giovanni Luca, Francesca Mancuso

**Affiliations:** 1 Department of Medicine and Surgery, University of Perugia, 06132 Perugia, Italy; iva.arato@unipg.it (I.A.); mario.calvitti@unipg.it (M.C.); eugeni.elena@gmail.com (E.E.); giovanni.luca@unipg.it (G.L.); 2 Department of Pharmaceutical Sciences, University of Perugia, 06132 Perugia, Italy; stefano.giovagnoli@unipg.it (S.G.); roscini.lu@gmail.com (L.R.); gianluigi.cardinali@unipg.it (G.C.); 3 International Biotechnological Center for Endocrine, Metabolic and Embryo-Reproductive Translational Research (CIRTEMER), Department of Medicine and Surgery, University of Perugia, 06132 Perugia, Italy; 4 Division of Medical Andrology and Endocrinology of Reproduction, Saint Mary Hospital, 05100 Terni, Italy

**Keywords:** Sertoli cells, *Candida tropicalis*, microparticles, drug delivery, nonprofessional tolerogenic

## Abstract

This study aimed to evaluate the intrinsic *in vitro* performance of naïve porcine prepubertal Sertoli cells (SCs) and SCs loaded with blank poly(lactic acid) microparticles (MP) or amphotericin B poly(lactic acid) microparticles (AmB-MP) against *Candida tropicalis*, a prevalent pathogenic non-*albicans* species. The objective was to assess their impact on biofilm formation and the cellular response mechanisms involved, building on previous findings that highlight SCs’ potential as anti-infective agents and drug carriers. Our results demonstrated that SCs successfully internalized *Candida tropicalis* while maintaining viability and exhibited a strong anti-infective effect, inhibiting biofilm formation by 70%. This inhibition increased to 80–90% when SCs were combined with AmB-MP. The interaction between SCs (both naïve and MP-loaded) and *Candida tropicalis* triggered the activation of MAPK, AKT, and NF-kB signaling pathways, leading to the upregulated expression of innate immune factors such as MHC-II, TLR-4, TGF-β, IDO, and β-defensin 123. These findings reinforce the role of SCs in infection control and drug delivery. Furthermore, their anti-infective and scavenging activity is linked to a tolerogenic phenotype, suggesting a potential dual therapeutic role at the host–pathogen interface.

## 1. Introduction

Cell intrinsic competencies and functions have inspired applications as therapeutic agents and drug delivery systems [1,2,3,4,5], with some clinical success [6,7,8,9,10]. Beyond concerns regarding the safety of this approach [11], the delivery of live cells may offer the advantage of coupling cell functionality with carrier capabilities.

Sertoli cells (hereinafter referred as SCs) possess the proper requisites for being transformed into therapeutic tools. In their role as the main hematotesticular barrier component and nourishing cell in the spermatogenic process, SCs present remarkable phagocytic capacity and immunocompetence [12,13,14,15,16]. Due to these properties, SCs have been used to treat diabetes [17,18] to expedite and improve pancreatic cell transplantation [19,20], to promote neural cell regeneration [21,22], and as immunomodulators to potentiate several other transplantation procedures [23,24]. SCs show a homing capacity that in part explains their targeting ability [25,26].

Moreover, SCs can respond to infection at the host–pathogen interface by regulating the body’s immune response as well as directly tackling microorganism invasion and proliferation [26,27,28,29,30]. The antimicrobial response of SCs is associated with the secretion of defensins in rodents and canines [27,28] and with the high-mobility group box chromosomal protein 1 in rat and human SCs [29].

Therefore, SCs not only serve as a physical barrier, but can also enable the modulation of the immune response by secreting trophic, anti-inflammatory, and immunomodulatory factors [30]. In particular, SCs have been found to be potentially useful as safe drug carriers, with action against inflammatory conditions and infections. Kumar et al. transplanted rat SCs, pre-loaded with curcumin containing chitosan nanoparticles, in a mouse model of acute pulmonary inflammation. SCs accumulated preferentially in the lungs with no immune complications or other side effects observed [25]. In another study, SCs’ phagocytic and antibacterial activities were exploited to formulate an SC-based drug delivery system [31]. The SCs loaded with microencapsulated antibiotic complexes showed good antibacterial activity over time and storage potential, presenting a basis for the development of novel cell-based drug delivery systems.

In support of this assumption, Arato et al. demonstrated that SCs, under effects of external stimuli in the form of polymeric microparticles and bacteria-derived endotoxins such as lipopolysaccharides (LPSs), exhibited a significant increase in the gene expression of several mediators, including β defensins, TGF-β1, IDO, MHCII, and ICAM1, suggesting the “role” of nonprofessional tolerogenic antigen-presenting cells in addition to their typical function in blood–testicular barrier cells [32].

These data supported the previous study that demonstrated how SCs could be turned into valuable drug delivery and antimicrobial agents, with the possibility of storage and cell banking [31].

A biofilm is a complex network of heterogeneous microbial communities, encased into a protective matrix mainly made of secreted polysaccharides and glycoproteins. This permits longer persistence in nosocomial environments compared to planktonic cells [33,34,35].

Biofilms are hard to eradicate and frequently a source of inconsistent therapeutic effects [36]. Antibiotic resistance results from the presence of persister cells with a slow metabolism and growth rate and a thick matrix, with low oxygen levels and inactivating enzymes that deplete drug action. Such conditions promote exacerbation, chronic infections, and reinfection [37,38,39]. Several strategies have been proposed to contrast biofilm formation methods with variable degrees of clinical success [40].

Therefore, there is a tremendous need for new strategies aimed at restoring host–pathogen fitness rather than aggressively tackling microbial populations with a paradigm shift towards a new concept of antibiotic therapy.

SCs may offer this possibility via their dual role as scavengers and immune regulators, as well as via their proven capability as delivery agents.

*Candida tropicalis (C. Tropicalis)* has been identified as the most prevalent pathogenic yeast species of the non-*albicans Candida* group. The presence of *C. tropicalis*-related candidiasis increased dramatically worldwide due to azole/amphotericin B cross-resistance, which elevated *C. tropicalis* to the alarming status of an emerging pathogen, also justifying the push for dedicated prospective studies [41].

Based on these premises, this work aimed to evaluate SCs’ interactions and inhibition effects on *C. tropicalis* biofilms. Fungal biofilms were placed in direct contact with naïve porcine prepubertal SCs or SCs preloaded with blank poly(lactic acid) or amphoterycin B poly(lactic acid) microparticles (MP and AmB-MP, respectively). The SCs response was characterized by addressing the activated mediators and identifying the underlying signaling pathways involved. This work further points to the potential dual benefits of SCs based treatments able to target the host–pathogen interface.

## 2. Materials and Methods

### 2.1. Preparation of MP and AmB-MP

Both blank MP and AmB-MP formulations were produced by a Mini Spray- Dryer Model B-290 (Büchi, Milan, Italy) starting from drug and polymer solutions. The polymer used was poly DL-lactide R203H (PLA, MW 20–30 kDa, Boehringer Ingelheim, Germany). Briefly, amphotericin B (AmB, Sigma–Aldrich, St. Louis, MO, USA) was solubilized in methanol (Carlo Erba Reagents, Milan, Italy) and mixed with a PLA solution in acetonitrile and the two solutions were mixed to achieve 2% *w*/*v* feedstock concentration and immediately sprayed. Blank MP were produced by directly spraying the PLA solution in acetonitrile. The instrumental parameters were set as follows: feed rate 2.4 mL/min, air flow rate 357 L/h, inlet temperature 75 °C [31,42]. The microparticles were harvested by a high efficiency cyclone (Buchi, Milan, Italy).

### 2.2. MP and AmB-MP Size, Morphology and Quantification

Blank MP and AmB-MP were analyzed dimensionally employing an Optical Particle Sizer ACCUSIZER C770 (PSS, Santa Barbara, CA, USA). A small fraction of the preparation was suspended in water, vortexed, and subjected to sonication. The size distribution was expressed as volume mean diameter (VMD) while the span parameter (size distribution) was calculated as follows (Equation (1)):

Span = d_(90)_−d_(10)_/d_(50)_ (1)

where d_(90)_, d_(10)_, and d_(50)_ are the diameters ≤ 90%, 10%, and 50% of the population distribution, respectively.

Blank MP and AmB-MP were morphologically characterized by Scanning Electron Microscopy (SEM) using a FEG LEO 1525 microscope (LEO Electron Microscopy Inc., New York, NY, USA). The acceleration potential voltage was maintained at 10 keV. Dry powders were placed onto carbon tape-coated aluminum stubs. The stubs were sputter-coated with chromium before imaging by a high-resolution sputter (Quorum Technologies, East Essex, UK). Coating was performed at 20 mA for 30 s [31].

The amount of AmB encapsulated in the MP was quantified by UV-vis spectrophotometry and HPLC method. For the first analysis, an Agilent 8453 (Agilent, Waldbronn, Germany) spectrophotometer was used at λmax = 406 nm upon calibration in the range 5–30 µg/mL in methanol (r^2^ = 0.99917). Samples were prepared by the extraction of the drug from the AmB-MP. Briefly, we weighed out a certain amount of AmB-MP and dispersed them in acetonitrile and properly diluted them in methanol before analysis. When required, samples were centrifuged (300× *g*, 5 min) to eliminate polymer flocculates. The amount of AmB encapsulated in MP was indicated as % drug content (%DC) according to Equation (2):

%DC = (µg AB/mg dry powder) × 100(2)

For the second quantification method, an isocratic HPLC method was employed by using a Portlab STAYER HPLC system equipped with UV detection, a parallel pump, and Triathlon autosampler (Portlab, Milan, Italy). The column used was Crom-Sil 120 Diol 5 µm 250 × 4.6 mm (Grace, Milan, Italy), equilibrated at 25 °C. As a mobile phase, we employed a solution of acetonitrile–0.05M perfluoropentanoic acid at 85:15 (Sigma Aldrich, Milan, Italy). This was prepared daily and degassed before use. Instrumental parameters were as follows: column temperature = 25 °C; flow rate = 1.00 mL/min. Detection was performed using UV-vis at 406 nm. A standard curve was built between 1.25 and 20 µg/mL (r^2^ = 0.9997).

### 2.3. In Vitro Drug Release

To evaluate the stability of the AmB-MP, we performed *in vitro* release assays for up to 24 h, suspending a weighed amount of AmB-MP in 10 mL of Hank’s Balanced Salt Solution medium (HBSS, Sigma Aldrich, Milan, Italy) at 37 °C. After the centrifugation of AmB-MP (300× *g*, 5 min), 500 μL of supernantant was withdrawn at established time points and replaced with an equal volume of fresh HBSS (Sigma Aldrich, Milan, Italy) medium. Samples were properly diluted in methanol and submitted to HPLC analysis as reported above. Drug release was calculated in terms of %*w*/*w* of AmB released.

### 2.4. Porcine Prepubertal SCs Isolation

Overall, 3 Danish Duroc prepubertal pigs, aged between 15 and 20 days, underwent bilateral orchidectomy under general anesthesia using ketamine (Ketavet 100; Intervet, Milan, Italy) at a dose of 40 mg/kg, and dexmedetomidine (Dexdomitor, Orion Corporation, Espoo, Finland) at a dose of 40 µg/kg. These pigs were used as donors for Sertoli cells (SCs). Specifically, pure prepubertal porcine SCs were isolated, characterized, and evaluated for their functional competence, following previously established protocols [43,44].

### 2.5. Uptake Process Evaluation

The uptake of AmB-MP at 3 × 10^5^ SCs /cm^2^, cell concentration established on our previously published data [45,46], was evaluated at four different concentrations—10 µg, 20 µg, 30 µg, and 40 µg/cm^2^—dispersed in HAM’S F-12 medium (Lonza, Verviers, Belgium) by exploiting the innate phagocytic capacity of SCs. Before loading, the exact amount of AmB-MP was weighed for all four concentrations, sterilized with UV light for 30 min, and suspended in 10 mL of HAM’S F-12 medium. Then, the four suspensions were sonicated and vortexed in the end to obtain a homogeneous dispersion by preventing the formation of lumps. Subsequently, the SCs were loaded and then incubated at 37 °C for 5 h [31]. Thereafter, the cell monolayer was detached by trypsin/ethylenediaminetetraacetic acid (EDTA) (Lonza, Verviers, Belgium) treatment at 37 °C for 8 min to promote the enzymatic reaction. After washing with 1 mL of HBSS (Sigma Aldrich, Milan, Italy), SCs viability was measured by an automated cell counter (INVITROGEN, Countess Automated Cell Counter, CA, USA) after trypan blue staining (Sigma–Aldrich, Milan, Italy) [47]. Finally, cell pellets were centrifuged at 1500× *g* for 6 min and stored at −80 °C.

Analysis was performed by extracting the drug from the pellets in methanol solution upon vortexing and sonication steps at room temperature (RT). After proper centrifugation (300× *g*, 5 min), the supernatants were recovered, properly diluted in methanol, and immediately submitted to HPLC analysis, as described above.

After determining the most suitable AmB-MP concentration, the uptake process was investigated over time at 5, 24, 72 h, and 7 days.

### 2.6. Biofilm Formation Protocol

The biofilm was prepared following Pierce’s Modified Protocol [48]. In particular, a preculture of the *C. tropicalis* CMC_2024 strain was prepared. We performed this by inoculating flasks containing YEPD (Yeast Extract 1%, Peptone 1%, Dextrose 2%—Biolife, Milan, Italy) + 0.05% chloramphenicol (Merck KGaA, Darmstadt, Germany) liquid medium with a loopful of cells from the stock cultures and incubated them overnight in an orbital shaker (150–180 rpm) at 30 °C. Cells were harvested from the liquid used for overnight growth by centrifugation (approximately 3000× *g* for 5 min at 4 °C); the supernatant was removed and the pellet was washed twice in sterile saline. The final pellet was resuspended in RPMI-1640 medium (Euroclone, Milan, Italy) that had been pre-warmed at 37 °C. From the resulting cell suspension, 1:100- and/or 1:1000-fold dilutions in the same medium were prepared and counted using a Thoma–Zeiss hemocytometer and a bright field microscope [Leica Microsystems— Buccinasco (Milan—Italy)] with a magnification of 400×. Alternatively, optical densities were recorded at 600 nm, and we took care to read the cell suspensions at OD_600_ within a range from 0.1 to 0.5 to guarantee the linearity between the cells and the optical density. The actual optical density was calculated by multiplying the dilution factor by the recorded OD_600_. After counting, the volumes needed to prepare a suspension of cells at a final density of 1.0 × 10^6^ cells/mL (OD_600_ = 0.1) in RPMI 1640 (Euroclone, Milan, Italy) were calculated.

After the seeding, the plates were covered with their original lid, sealed, and incubated statically for 2 h at 37 °C. After biofilm formation, the medium was carefully aspirated so as not to touch and disrupt the biofilm, which was washed thrice with sterile saline to remove planktonic and/or non-adherent cells that remained in the wells. After each wash, the plates were drained using a sterile pipette to remove any residual saline. Then, biofilms were ready to be processed via antifungal susceptibility testing assays described in Supplementary Material. To assess the starting amount of formed biofilm required in order to perform co-culturing with Sertoli cells [timing~100 h], the OD_405_ was read by a TECAN Infinite 200 pro series plate reader (Tecan Trading AG, Zurich, Switzerland).

### 2.7. Dose–Response Evaluation

Based on the results of pilot experiments described in Supplementary Material, in which the biofilm mass was estimated based on the spectrophotometric measurement of the (2,3,5-triphenyltetrazolium chloride) (TTC) reduction at 405 nm according to the methods of Pierce et al. [48], we observed that the best experimental condition in which to perform the experiments was that with the SCs placed in direct contact with the biofilm of *C. tropicalis* (Figure S1, Figure S2 and Table S1 in Supplementary Material). To evaluate the minimum inhibitory concentration (MIC) of SCs able to inhibit the visible growth of *C. tropicalis,* 1 × 10^5^ SCs /cm^2^, 2 × 10^5^ SCs /cm^2^, and 3 × 10^5^ SCs /cm^2^ loaded with blank MP and AmB-MP, at a concentration of 30 µg/cm^2^, were placed in direct contact with the biofilm at 37 °C for 24, 48, 72, and 96 h. At each endpoint and for all experimental conditions, optical microscopy photographs were performed to demonstrate the optimal state of integrity of the SC monolayer and the inhibitory effect on biofilm growth (Figures 5–7).

### 2.8. Experimental Design

According to the results obtained by the dose–response evaluation, the concentration of 1 × 10^5^ SCs/cm^2^ was established as MIC and employed in the following experimental conditions and evaluated at the time points of 24, 48, 72, and 96 h:

- Control: unexposed SCs
- SCs + *C. tropicalis*
- SCs + AmB-MP
- SCs + AmB-MP SCs + *C. tropicalis*
- SCs + MP
- SCs + MP + *C. tropicalis*

Samples required to perform all necessary analyses were collected at each experimental time point.

### 2.9. RT-PCR Analysis

At the same time, total RNA was isolated following the method described previously [49]. In brief, RNA was extracted from the scraped monolayers using an RNA purification kit (Versagene RNA Cell Kit, Gentra Systems, Minneapolis, MN, USA) and quantified by measuring the optical density at 260 nm. Then, 2 µg of total RNA was used for reverse transcription (RT) in a final volume of 20 µL (SuperScript™ VILO™ cDNA Synthesis Kit, ThermoFisher Scientific, Waltham, MA, USA). Real-time PCR was performed using 1 µL of cDNA from the RT reaction and SYBR Green (Stratagene, Amsterdam, The Netherlands). The primer sequences for each gene are listed in Table 1. Real-time PCR was conducted in an Mx3000P cycler (Stratagene, Amsterdam, The Netherlands), using FAM for detection and ROX as a reference dye. One-step PCR was performed in 25 µL of Brilliant SYBR^®^ Green QPCR Master Mix (Stratagene, Amsterdam, The Netherlands) following the manufacturer’s instructions. Product formation was monitored continuously using the fluorescent double-stranded DNA binding dye SYBR^®^ Green at each annealing step. The relative expression level of the housekeeping gene β-actin was used to normalize the expression of marker genes in each sample. Immediately after PCR, a melting curve was generated by increasing the incubation temperature from 55–95 °C to confirm amplification specificity. The results were presented as fold changes relative to the control values of SCs alone, and all data were calculated using the MxPro QPCR Software, version 4.10 (Stratagene, Amsterdam, The Netherlands).

### 2.10. Western Blot (WB) Analysis

Western blotting (WB) analysis was performed to investigate the activated mediators and identify the signaling pathways involved in the experimental setup.

Specifically, SCs monolayers were scraped to obtain cell extracts, which were then resuspended in 100 µL of radioimmunoprecipitation assay lysis buffer (RIPA buffer, Santa Cruz Biotechnology Inc., Santa Cruz, CA, USA). After centrifugation at 1000× *g* (Eppendorf, Enfield, CT, USA) for 10 min, the supernatant was collected, and total protein levels were quantified using the Bradford method [50]. Sample aliquots were stored at −20 °C for later WB analysis.

Cell extracts with equal protein amounts (70 µg protein per lane) were separated using 4–15% SDS-PAGE and transferred to nitrocellulose membranes (BioRad, Hercules, CA, USA). The membranes were incubated overnight in a buffer containing 10 mM TRIS, 0.5 M NaCl, and 1% (*v*/*v*) Tween 20 (Sigma-Aldrich, St. Louis, MO, USA) with following primary antibodies: mouse anti-phospho-ERK1/2 (Millipore, Burlington, MA, USA; dilution factor 1:100), rabbit anti-phospho-JNK (Millipore, Burlington, MA, USA; dilution factor 1:500), rabbit anti-phospho-Akt (Cell Signaling, Danvers, MA, USA; dilution factor 1:1000), rabbit anti-pNF-kB (Cell Signaling, Danvers, MA, USA; dilution factor 1:1000), rabbit anti-DEFB123 (STJ196187, St John’s Laboratory, London, UK; dilution factor 1:500), mouse anti-IDO (Millipore, Burlington, MA, USA; dilution factor 1:500), and mouse anti-GAPDH (Sigma-Aldrich, St. Louis, MO, USA; dilution factor 1:100). The membranes were then incubated for an additional 60 min with horseradish peroxidase-conjugated (HRP) secondary antibodies: anti-rabbit (Sigma-Aldrich, St. Louis, MO, USA; dilution factor 1:5000) and/or anti-mouse (Santa Cruz Biotechnology Inc., Santa Cruz, CA, USA; dilution factor 1:5000). The bands were visualized using enhanced chemiluminescence (ECL) [51].

### 2.11. Statistical Analysis

Normality analysis was conducted using the Shapiro–Wilk test, and statistical comparisons were performed using one-way ANOVA followed by Tukey’s HSD post hoc test (SigmaStat 4.0 software, Systat Software Inc., San Jose, CA, USA). The results are presented as the means ± S.E.M. of three independent experiments, each performed in triplicate. Differences were considered statistically significant at * *p* < 0.05 and ** *p* < 0.001 when compared to unexposed SCs.

### 2.12. Ethics Approval

This study was performed in line with the principles of the Declaration of Helsinki. Approval was granted by the Ethics Committee of the University of Perugia and the Italian Ministry of Health (468/2017-PR, 6 June 2017)

## 3. Results

### 3.1. MP and AmB-MP Characterization

#### 3.1.1. Size and Morphology

**Figure 1:**
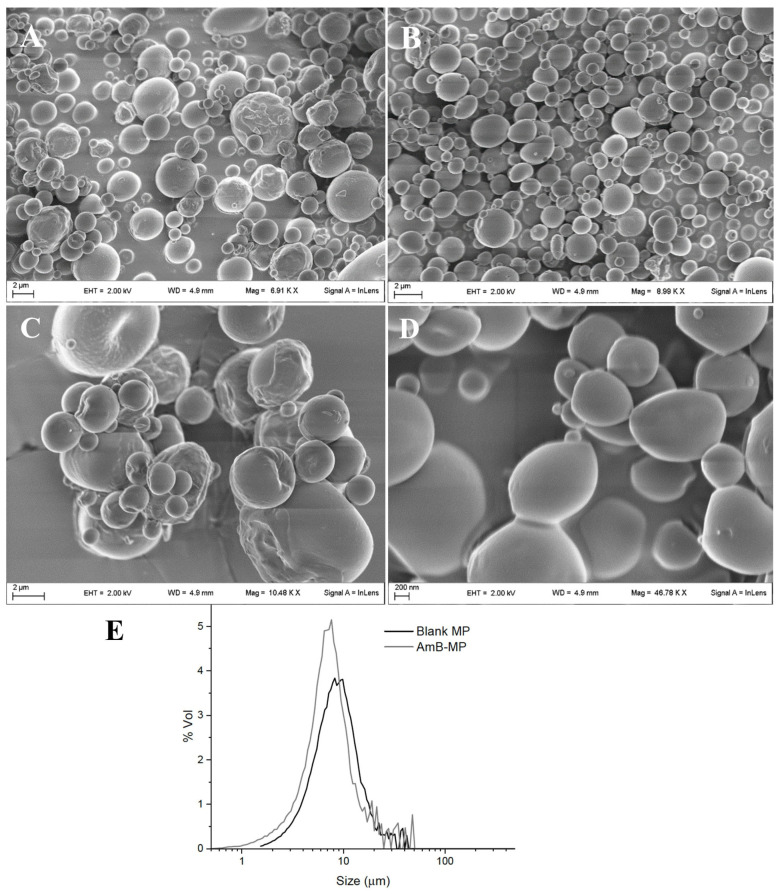
MP characterization: SEM analysis showed that (**A**,**C**) both blank MP and (**B**,**D**) AmB-MP presented similar spherical shapes with a nearly smooth surface. (**E**) Granulometric analysis revealed that AmB loading was uninfluential on the size and size distribution of blank MP and AmB-MP, with a mean volumetric diameter (MVD) around 8.1 and 7.2 μm and a span from 1.8 to 1.6 μm, respectively.

In our study, SEM analysis showed that both blank MP and AmB-MP, produced by spray-drying, presented a similar spherical shape with a nearly smooth surface (Figure 1A,B). Granulometric analysis revealed that AmB loading was uninfluential on size distribution, with a VMD around 8.1 and 7.2 μm and a span of 1.8 and 1.6 for blank MP and AmB-MP, respectively (Figure 1C,D).

The dimensions observed by SEM were consistent with the dimensional distribution evaluated by granulometry and, hence, were compatible with possible SCs uptake since the cells were found capable of phagocytizing particles with a size of 10–20 µm as well (Figures S1 and S3 in Supplementary Material), [31].

#### 3.1.2. AmB Analysis in Formulation and *In Vitro* Drug Release

**Figure 2:**
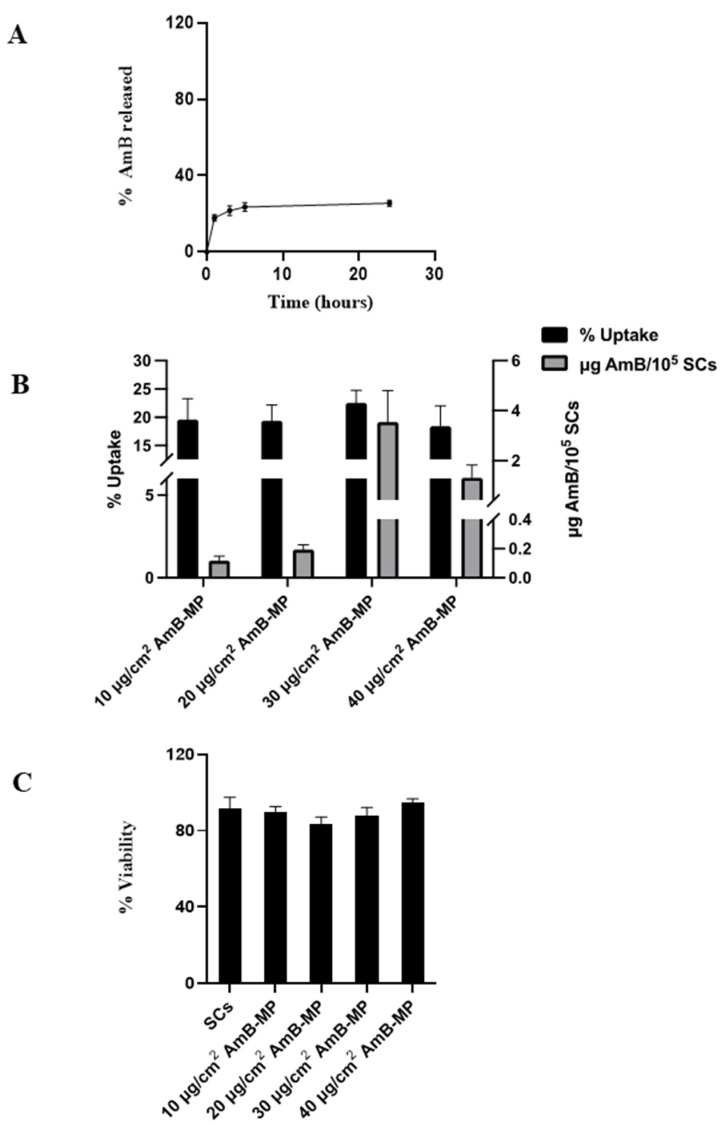
The evaluation of the *in vitro* drug release and uptake process. (**A**) To evaluate the stability of the AmB-MP, we performed *in vitro* release assays for up to 24 h, suspending a weighed amount of AmB-MP in 10 mL of Hank’s Balanced Salt Solution medium at 37 °C, and assessing the drug release in the supernatant by HPLC analysis, as reported in the text. (**B**) The evaluation of the uptake process expressed as a percentage and/or µg AmB/10^5^ SCs for 3 × 10^5^/cm^2^ SCs loaded with 10, 20, 30, and 40 µg/cm^2^ of AmB-MP. (**C**) SC viability was evaluated by tripan blue staining (see text for major details) at 5 h after treatment with 10, 20, 30 and 40 µg/cm^2^ of AmB-MP.

Based on our data, the percentage of AmB released over the first 5 h was about 23.59 ± 2.22% (Figure 2A), and no further release occurred up to 24 h (25.5 ± 1.59%). This value was considered suitable for effective uptake by cells.

In fact, the low initial release of AmB should ensure limited loss of the drug in the time required for the uptake of AmB-MP. This should have reached its plateau at 5 h without adversely affecting cell morphology and viability, as previously reported [31].

### 3.2. Uptake Process Evaluation and SC Viability

To investigate the interaction between AmB-MP and SCs and the possible cytotoxic effect, 3 × 10^5^ SCs/cm^2^ were exposed to four different AmB-MP concentrations for 5 h (Figure 2B,C). As shown in Figure 2C, the best concentration of AmB-MP was 30 µg/cm^2^, which produced an uptake of 22.53 ± 2.22% and maximized drug levels in cells, achieving 3.54 ± 1.25 µg AmB/10^5^ SCs.

This concentration resulted in non-toxic outcomes, as demonstrated in Figure 2C, in which the SCs viability remained always above 85%, thus demonstrating that the selected uptake conditions did not undermine SCs integrity.

Successively, this process was followed up fort to 7 days to evaluate behavior over time.

**Figure 3:**
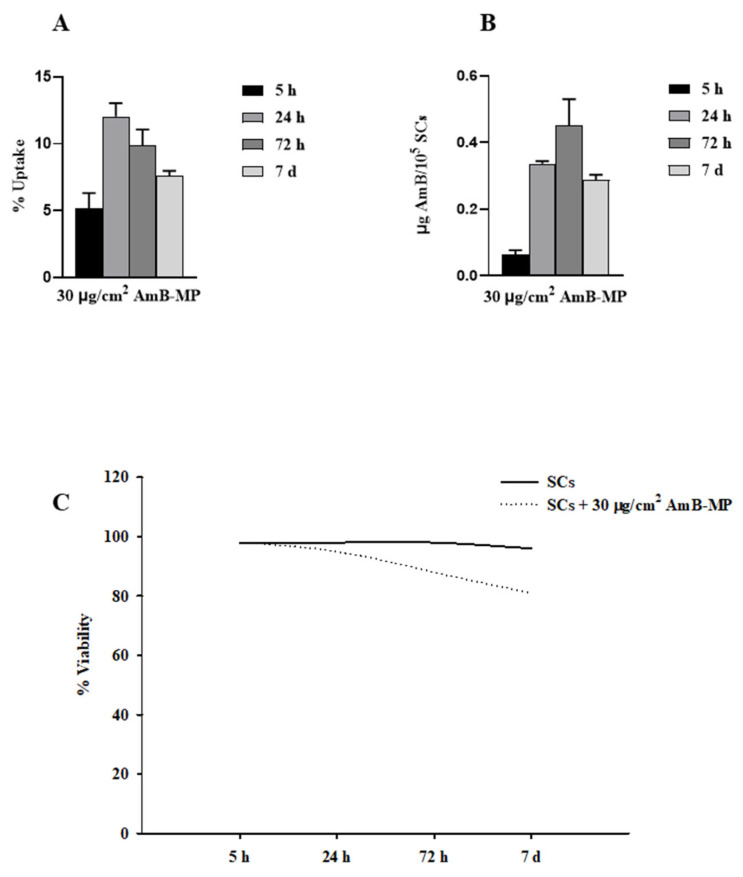
The evaluation of the uptake process over time. (**A**) The time trend uptake by 3 × 10^5^ SCs /cm^2^ loaded with AmB-MP at 30 µg/cm^2^ concentration up to 7 days. (**B**) The time trend of drug content for 3 × 10^5^ SCs /cm^2^ loaded with AmB-MP at a 30 µg/cm^2^ concentration over 7 days. (**C**) The representation of the relative SCs viability rate up to 7 days during the uptake process previously described.

As can be seen in Figure 3A, the uptake process of 30 µg/cm^2^ of AmB-MP rose by 234.2% compared to the initial state (T0 = 5 h) at 24 h, remaining above the T0 until day 7.

This finding was further confirmed by the increase in the amount of drug in the cells, that remained above T0 until day 7 (Figure 3B).

Interestingly, the cell viability of SCs loaded with the AmB-MP remained above 80% compared to unexposed SCs up to 7 days of incubation (Figure 3C).

### 3.3. SCs Anti-Infective Action

**Figure 4:**
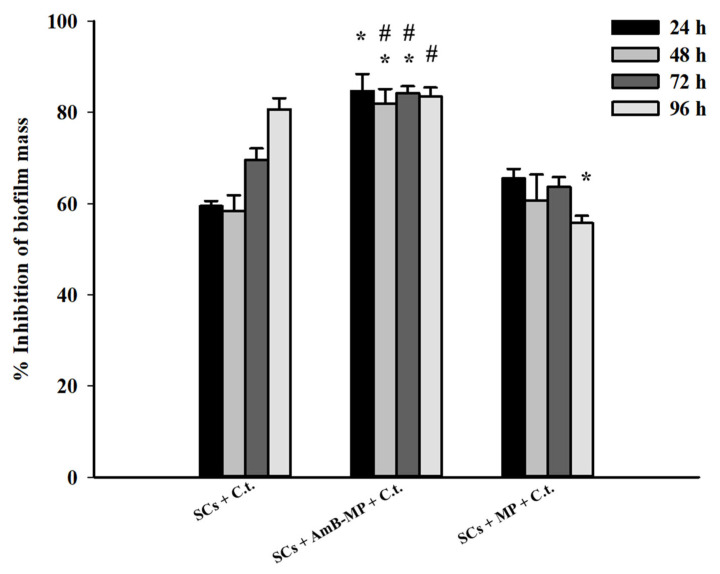
Percentage of biofilm mass reduction displayed by *C. tropicalis* following treatment with naïve SCs, SCs loaded with AmB-MP, and blank MP. SC concentration = 1 × 10^5^/cm^2^. C.t. (*C. tropicalis*). The results are presented as mean + SEM. * *p* < 0.05 with respect to SCs + *C. tropicalis* and # *p* < 0.05 with respect to SCs + MP of three independent experiments, each performed in triplicate.

The value of 1 × 10^5^ SCs /cm^2^ (MIC) induced the statistically significant inhibition of *C. tropicalis*, with more than 70% inhibition at 96 h (either as naïve SCs or plus blank MP) with a pick of more than 80% of mass reduction, obtained upon treatment with AmB-MP-loaded SCs (Figure 4 and Supplementary Figure S4).

**Figure 5:**
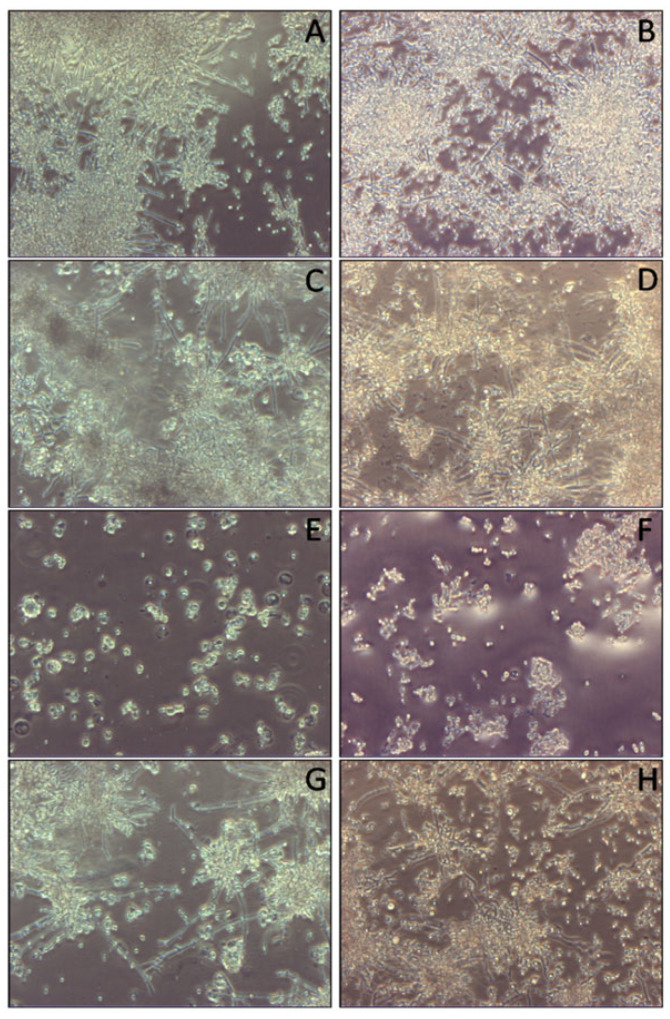
Light microscopy images of biofilm mass reduction exhibited by *C. tropicalis* following treatment with naïve SCs, SCs loaded with AmB-MP, and blank MP at the concentration of 1 × 10^5^/cm^2^. (**A**) Cell cultures of *C. tropicalis* alone at 24 h (hours) and (**B**) 96 h (**C**) SCs + *C. tropicalis* at 24 h and (**D**) 96 h. (**E**) SCs + AmB-MP + *C. tropicalis* at 24 h and (**F**) 96 h. (**G**) SCs + MP + *C. tropicalis* at 24 h and (**H**) 96 h.

These data were confirmed by optical microscopy at 24 and 96 h, which macroscopically showed a significant decrease in biofilm mass with SCs + *C. tropicalis* at 24 h (Figure 5C) and 96 h (Figure 5D), a significant decrease in biofilm mass with SCs + AmB-MP + *C. tropicalis* at 24 h (Figure 5E) and 96 h (Figure 5F), and a significant decrease in biofilm mass with SCs + MP + *C. tropicalis* at 24 h (Figure 5G) and 96 h (Figure 5H) compared to *C. tropicalis* alone at 24 h (Figure 5A) and 96 h (Figure 5B).

As expected, the *C. tropicalis* biofilm inhibition effect was enhanced by loading SCs with AmB-MP and, as is worthy of note, even with blank MP. This induced the statistically significant inhibition of *C. tropicalis* biofilm mass, limited by more than 70% compared to *C. tropicalis* alone.

**Figure 6:**
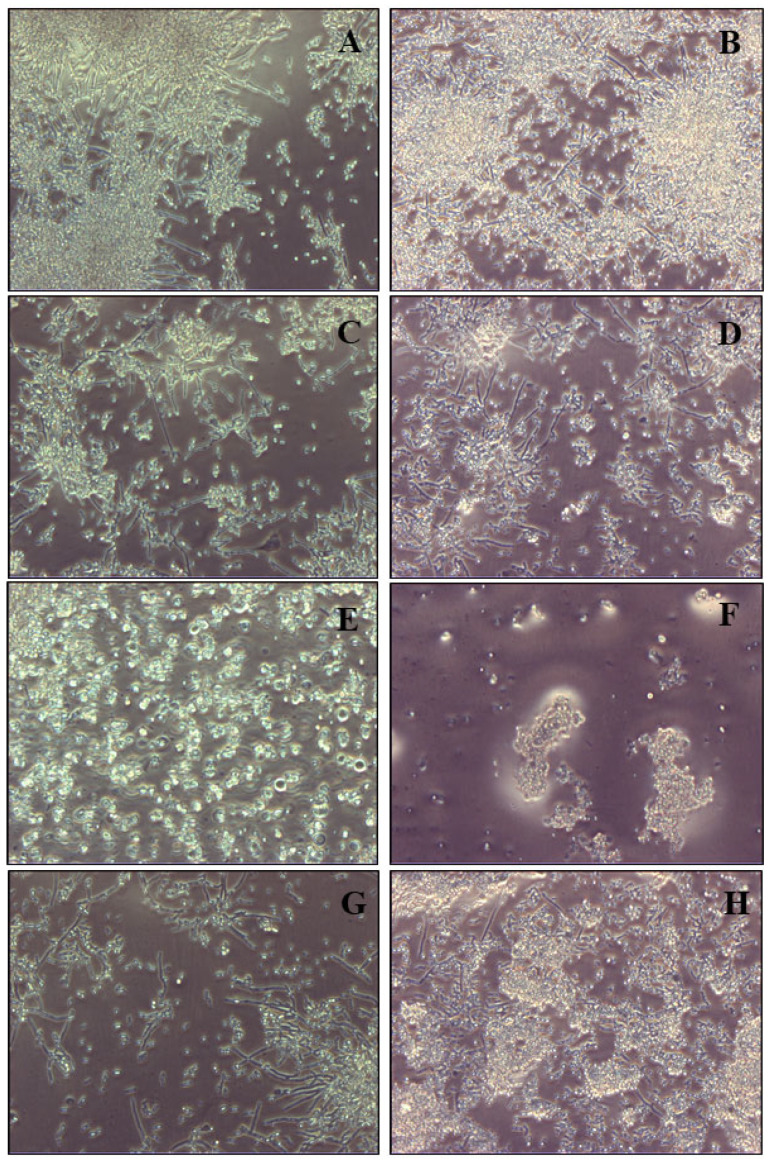
Light microscope images of biofilm mass reduction of *C. tropicalis* following treatment with naïve SCs, SCs loaded with AmB-MP and blank MP at the concentration of 2 × 10^5^/cm^2^. (**A**) Cell cultures of *C. tropicalis* alone at 24 h and (**B**) 96 h. (**C**) SCs + *C. tropicalis* at 24 h and (**D**) 96 h. (**E**) SCs + AmB-MP + *C. tropicalis* at 24 h and (**F**) 96 h. (**G**) SCs + MP + *C. tropicalis* at 24 h and (**H**) 96 h.

**Figure 7:**
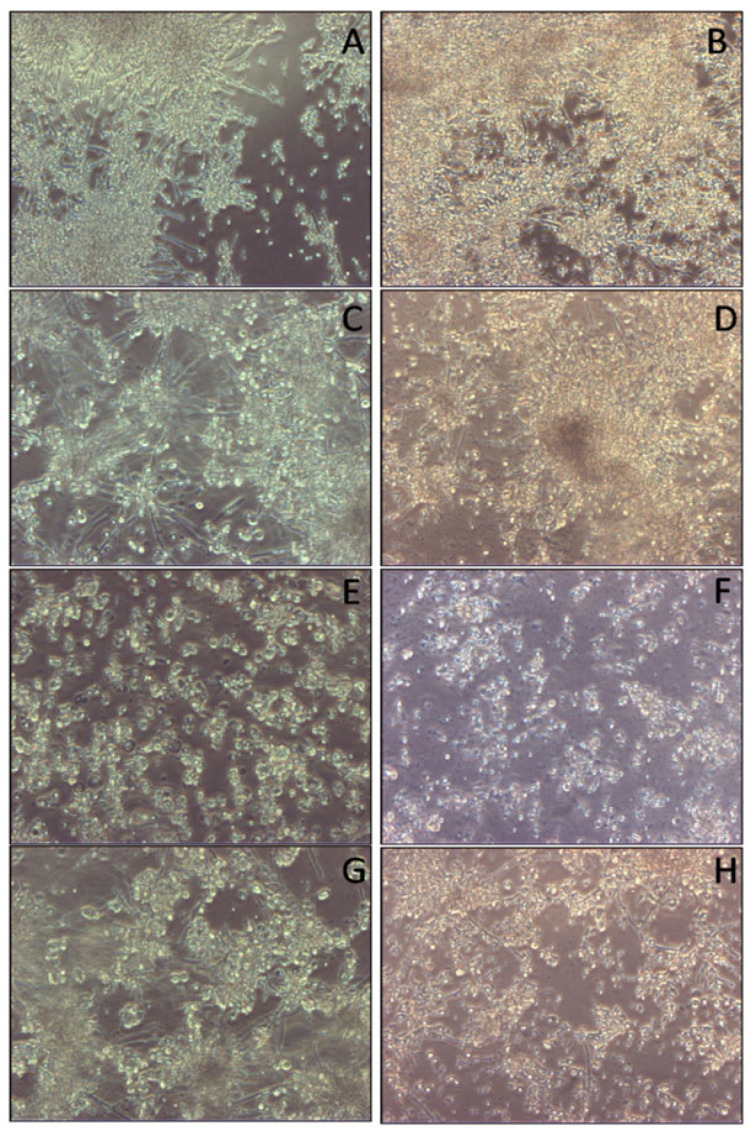
Light microscope images of biofilm mass reduction of *C. tropicalis* following treatment with naïve SCs, SCs loaded with AmB-MP and blank MP at the concentration of 3 × 10^5^/cm^2^. (**A**) Cell cultures of *C. tropicalis* alone at 24 h and (**B**) 96 h. (**C**) SCs + *C. tropicalis* at 24 h and (**D**) 96 h. (**E**) SCs + AmB-MP + *C. tropicalis* at 24 h and (**F**) 96 h. (**G**) SCs + MP + *C. tropicalis* at 24 h and (**H**) 96 h.

Of course, these results were even more noticeable at the concentrations of 2 × 10^5^ and 3 × 10^5^ SCs /cm^2^ (Figure 6 and Figure 7).

### 3.4. SCs Innate Immunity Response

**Figure 8:**
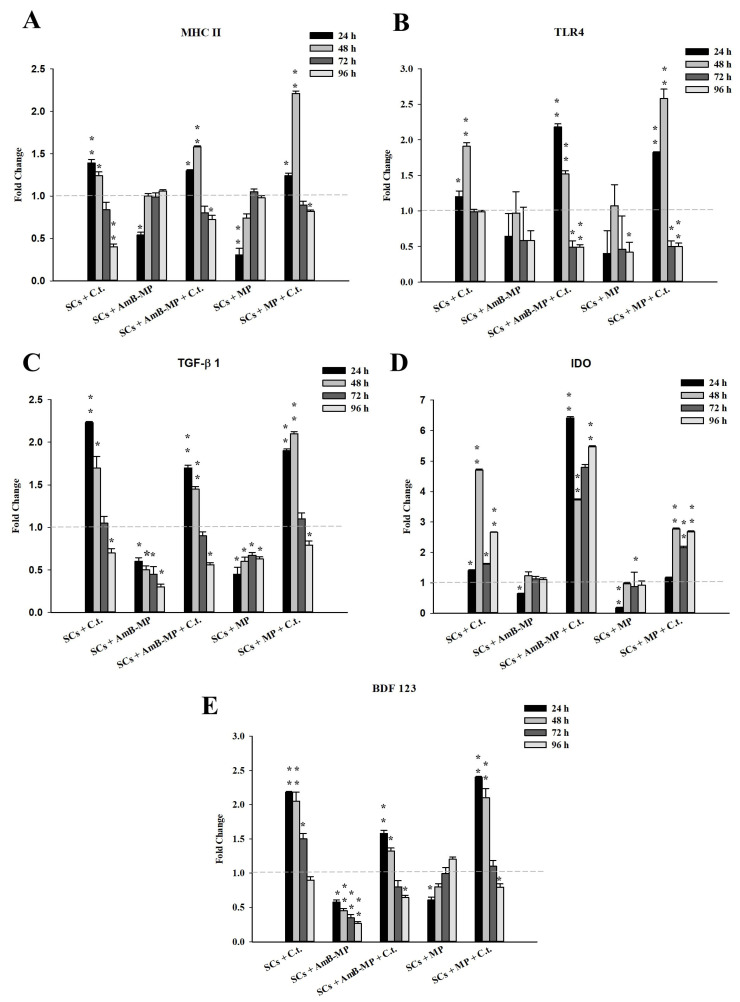
Real-time PCR analysis. (**A**) The gene expression of MHC II, (**B**) TLR4, (**C**) TGF-β1, (**D**) IDO, and (**E**) BDF 123 in the different culture conditions. SCs + *C. tropicalis*, SCs + AmB-MP, SCs + AmB-MP + *C. tropicalis*, SCs + MP, SCs + MP + *C. tropicalis*. C.t. (*C. tropicalis*). The results presented as mean + SEM. (* *p* < 0.05 and ** *p* < 0.001, respect to unexposed SCs (gray dotted line) of three independent experiments, each performed in triplicate).

**Figure 9:**
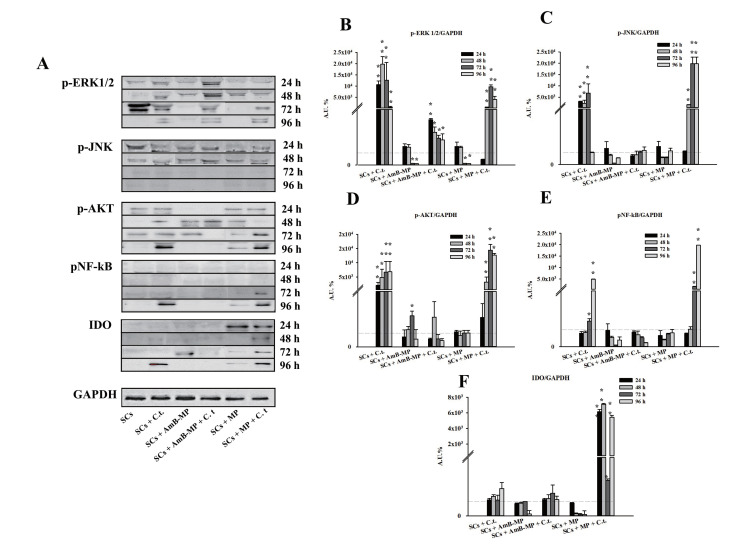
WB analysis. (**A**) Immunoblots of p-ERK1/2, p-JNK, p-AKT and pNF-kB in different culture conditions. SCs + *C. tropicalis*, SCs + AmB-MP, SCs + AmB-MP + *C. tropicalis*, SCs + MP, SCs + MP + *C. tropicalis*. C.t. (*C. tropicalis*). Densitometric analysis of the protein bands of (**B**) p-ERK1/2/GAPDH, (**C**) p-JNK/GAPDH, (**D**) p-AKT/GAPDH, (**E**) NF-kB/GAPDH, (**F**) IDO. The results presented as mean + SEM. (* *p* < 0.05 and ** *p* < 0.001, respect to unexposed SCs (gray dotted line) of three independent experiments, each performed in triplicate).

We focused our attention on the gene expression analysis of MHC-II, TLR4, TGF-β1, IDO, and β-defensin 123 (BDF123). Up to 48 h, we observed a significant increase in the gene expression of the innate response in all conditions under exposure to *C. tropicalis* (naïve SCs, and SCs loaded with blank MP and AmB-MP), contrary to what has been observed in both blank MP and AmB-MP SCs loaded alone (Figure 8A–E). From 72 h until the end of exposure, the gene expression of these molecules tended to be significantly reduced, except for the IDO messenger (Figure 8D) and molecule (Figure 9F), and BDF123 protein secretion in the medium (Supplementary Figure S5), compared with unexposed SCs, as confirmed by the Western Blotting analysis.

### 3.5. MAPK, AKT, and NF-kB Signal Pathways

We analyzed the signaling proteins MAPK and NF-kB, which orchestrate the effector response via the production of proinflammatory mediators and promote cell survival (Figure 9A).

At 24 h and up to 96 h, we observed that p-ERK1/2 levels were significantly increased in naïve SCs, SCs loaded with blank MP, and AmB-MP exposed to *C.tropicalis* compared to unexposed SCs (Figure 9B). p-JNK levels were significantly increased, up to 72 h, in naïve SCs + *C. tropicalis* and SCs loaded with blank MP + *C. tropicalis* (Figure 9C).

Then, throughout the whole experiment, p-AKT levels were only significantly increased in naïve SCs and SCs loaded with blank MP exposed to *C. tropicalis,* compared to unexposed SCs (Figure 9D).

In addition, we analyzed the activation of NF—kB by the phosphorylation of NF-kB p65 (p-NF-kB). Throughout the whole experiment, levels of p-NF-kB were only statistically increased in naïve SCs and blank MP SCs loaded under exposure to *C. tropicalis* with respect to unexposed SCs (Figure 9E).

## 4. Discussion

In recent years, poly(lactic acid) (PLA) microparticles have been intensively investigated in order to achieve the best results in terms of controlled and localized *in vivo* drug release, which is a major challenge in developing safe therapy [52].

Polyesters have been used in the realization of several depots, approved by the Food and Drug Administration, and validated in phase 3 clinical protocols (Lupron^®^, https://www.clinicaltrials.gov/ct2/show/NCT00660010, accessed on 1 January 2024; Risperdal^®^, https://clinicaltrials.gov/ct2/show/NCT03160521, accessed on 1 January 2024), proving to be safe as adjuvants.

In our study, we selected microparticles in the place of nanoparticles for the SCs loading process to maximize the drug content. In fact, the amount of drug encapsulated in the AmB-MP, determined by UV-vis spectrophotometry, resulted in a mean DC of 15.8% which can be considered a good outcome [53].

Specifically, in the present work, by investigating the intrinsic *in vitro* performances of naïve porcine prepubertal SCs or SCs loaded with MP and AmB-MP against *C. tropicalis*, we demonstrated the SCs’ ability to internalize and inhibit *C. tropicalis* cells, maintaining viability (Supplementary Figures S3 and S4), and confirmed SCs’ anti-infective action, with the notable 70% inhibition of *C. tropicalis* biofilms compared to *C. tropicalis* alone, which grew to 80–90% in synergy with AmB-MP (Figure 4).

These data strengthen the concept that SCs display endocytotic capacity and, furthermore, demonstrate the feasibility of microparticle loading procedures.

Tests on *C. tropicalis* biofilms clearly confirmed the inhibitory effect of naïve SCs in each condition evaluated during our experimental design. This led to the proposal of previously unknown SC antifungal activity, aside from the well-known antibacterial activity of SCs. It was principally ascribed to the secretion of defensins in rodents, canines, and pig [27,28,31] and to presence of the high-mobility group box chromosomal protein 1 in rat and human SCs [29].

In addition, we observed that the interaction between naïve SCs or SCs pre-loaded with MP and *C. tropicalis* induced the increased gene expression of innate immunity factors, including MHC-II, TLR-4, TGF-β, IDO, and β-defensin 123 (Figure 8, Figure 9F and Supplementary Figure S5, respectively).

Innate immune responsiveness to pathogens is usually mediated by pattern recognition receptors (PRRs) that recognize specific pathogen-associated molecular patterns (PAMPs), thus activating intracellular signaling pathways to mediate their effects, such as the secretion of cytokines, the activation of inflammatory mediators, and the enhanced phagocytosis of pathogens. For example, in myeloid cells, several different PRRs, such as mannose receptor (MR), Toll-like receptor (TLR)2, TLR4, and dectin-1, have been associated with the recognition of *C. albicans* and other fungi [54].

It has also been reported that, an essential role in the development of optimal protective immunity against fungal diseases is fulfilled by MHCII, a well-known transmembrane protein expressed in antigen presenting cells (APCs), whose main function is to present processed antigens, derived primarily from exogenous sources, to CD4(+) T-lymphocytes, as a prerequisite for their activation, clonal expansion, and differentiation into effector cells [55].

Moreover, the balance between proinflammatory and anti-inflammatory signals is a prerequisite for successful host–pathogen interaction in fungal infections [56,57]. Although several cell types contribute to the regulation of immune responses, regulatory T lymphocytes producing IL-10 (T reg), with tolerogenic activity, have been described in fungal infections of both mice [58,59,60,61] and humans [62]. The capacity of Treg cells to inhibit components of innate and adaptive immunity is pivotal in their regulatory function and has led to the concept of “protective tolerance”, which implies that a host’s immune defense may be adequate for protection without necessarily eliminating fungal pathogens that would impair immune memory or cause an unacceptable level of tissue damage [56].

De Luca et al. in an *in vitro* model of infection against *C. albicans,* demonstrated that indoleamine-2,3-dioxygenase (IDO) plays a key role in the immuno-regulatory mechanism of infection, controlling the balance between Th cell subsets and Treg cells [63,64,65,66,67]. Fallarino et al., in 2009, demonstrated IDO’s expression in SCs via a TGFβ1-mediated IDO-dependent mechanism for the first time [68].

In particular, β-defensins, a major group of mammalian antimicrobial peptides, are expressed in SCs and represent one of the earliest mediators of the host’s defense in humans and animals. In fact, both α- and β-defensins are actively expressed during the hyphal transition of *C. albicans* and concur with other immune components in fighting against this pathogen. This mostly occurs at mucosal and epithelial barriers since they are able to discriminate host cells from fungi: defensins are able to recognize the pathogen cell wall (different in composition from the human ones) and to disrupt it through membrane permeabilization [27].

As previously reported [32], upon combined blank MP and LPS stimulation, SCs “switch” their role from typical cells of the blood testicular barrier to nonprofessional tolerogenic antigen-presenting cells, as demonstrated by the significant increase in the gene expression of innate immunity response mediators. This behavior was also confirmed in this study and proved that the upregulation of such immunomodulatory factors was induced by the presence of *C. tropicalis* at the early infection stages and then reduced following the successful control of infective states.

Moreover, our study confirmed the central role of tolerance at the fungus/host interface and emphasized the role of tryptophan catabolism in the tolerant state, providing significant insights into how microbial infections might be managed and controlled [67].

Aiming to better understand the inflammatory signaling cascades triggered after *C. tropicalis* infection and how these pathways correlate with the different infection profiles observed, we analyzed the signaling proteins MAPK and NF-kB, which orchestrate the effector response via the production of proinflammatory mediators [69,70,71] and promote cell survival [72,73].

In an infection model of oral epithelial cells with *C. albicans,* Moyes et al. described the critical role played by ERK1/2 in the control and resolution of inflammatory responses, ensuring both an optimal and tightly controlled immune response to *C. albicans* and the rapid deactivation of potential deleterious p38- and JNK-mediated inflammatory responses.

From 24 to 96 h, in our study, we similarly observed that p-ERK1/2 levels were significantly increased in naïve SCs, SCs loaded with blank MP, and AmB-MP exposed to *C. tropicalis*, compared to the unexposed SCs [74,75].

Then, we analyzed the activation of NF—kB by the phosphorylation of NF-kB p65 (p-NF-kB) given that ERK1/2 acts upstream and can activate this transcription factor, which is involved in the production of proinflammatory mediators [76]. Throughout the whole experiment, levels of p-NF-kB were only statistically increased in naïve SCs and blank MP SCs, loaded under exposure to *C. tropicalis,* with respect to unexposed SCs.

These data would seem to suggest that the presence of AmB inhibited the activation of the proinflammatory NF-kB signaling pathway and maintained the p-ERK signaling pathway, as a threshold level of activation for the other signaling pathways was not reached, and the fungus was regarded as “nondangerous”. In contrast, in naïve SCs and SCs loaded with MP, the infection with *C. tropicalis* led to the activation of NF-kB, ERK1/2, and JNK pathways, which orchestrated the effector response, thus confirming data reported in *C. albicans.*

In addition, throughout the whole experiment, p-AKT levels were significantly increased in naïve SCs and SCs loaded with blank MP exposed to *C. tropicalis* compared to unexposed SCs. These data would seem to agree with the literature data that reported, in an oral epithelial cell model of infection with *C. albicans*, the activation of PI3K/Akt signaling in addition to MAPK and NF-kB signaling, as the major epithelial response pathways against this fungus, playing a key role in protecting epithelial cells from *C. albicans*-induced damage. Once again, we can speculate that we did not observe a significant activation of the PI3K/AKT pathway in SCs loaded with AmB-MP because there were no potential deleterious p38- and JNK-mediated inflammatory responses.

## 5. Conclusions

Our study demonstrated that naïve SCs showed a high inhibition capacity of *C. tropicalis* biofilm, reaching values above 70% and maintaining them over time. Evident synergy was recorded when the SCs were loaded with the antifungal drug, bringing the inhibition values above 80–90%. Moreover, we observed that the interaction between SCs with *C. tropicalis*, alone or in conjunction with MP and AmB-MP, induced many typical features of non-professional dendritic cells, which led to the increased gene expression of important factors involved in innate immunity (MHC-II, TLR-4, TGF-β, IDO, β-defensin 123) and the activation of important signal transduction pathways such as MAPK, NF-kB, and AKT.

Our study demonstrated the potential therapeutic efficacy of SCs against *C. tropicalis*-related candidiasis and their abilities to contrast biofilm formation, further enhanced by their proven capability as drug carriers. Finally, we reported how the strategy of SCs was to establish a state of tolerance at the fungus/host interface rather than aggressively tackling microbial populations and provided significant insights into how microbial infections might be managed and controlled, thus proposing a new concept in antibiotic therapy.

## Figures and Tables

**Table 1:** Primer sequences for PCR analyses.

Gene	Forward	Reverse	$T_{a}$
β-actin	ATGGTGGGTATGGGTCAGAA	CTTCTCCATGTCGTCCCAGT	56 °C
MHCII	GACCAGATGAGGTTATTGG	GGTCCTGTAGTTGTGTCT	56 °C
IDO	ATGAAGGCGTTTGGGACACC	GAGGAATCCAGCAGCAGAGC	56 °C
TGF1β	GCCCTGGACACCAACTATTGC	GCTGCACTTGCAGGAGCGCAC	56 °C
TLR-4	CTTCACTACAGAGACTTCA	ACAATAACCTTCCGACTT	56 °C
BDF123	GAGTGCGTTGGGAAGATG	TCGGTATGTACTTGGGATGT	56 °C

## Data Availability

The original contributions presented in the study are included in the article and Supplementary Materials; further inquiries can be directed to the corresponding author.

## Supplementary Materials

The following supporting information can be downloaded at: https://www.mdpi.com/article/10.3390/cells14070495/s1, Figure S1: Morphological characterization.; Figure S2: Biofilm inhibition test.; Figure S3: Representative TEM images of SCs cultures alone and in presence of *C. tropicalis*; Figure S4: Metabolic inhibition of *C. tropicalis*; Figure S5: BDF123 secretion; Table S1: Experimental scheme of *C. tropicalis* biofilm inhibition test. References [26,51,77,78,79,80] are cited in the supplementary material.
