# Supplementary material for: Exploring Sertoli Cells’ Innate Bulwark Role Against Infections: In Vitro Performances on Candida tropicalis Biofilms
Source: Cells. 2025 Mar 26;14(7):495. doi: 10.3390/cells14070495 (PMC11988068; doi:10.3390/cells14070495)
Supplement: Supplementary file 1 [file cells-14-00495-s001.zip › cells-3468394-supplementary-edit.pdf]

Supplementary materials

Exploring the Sertoli cells innate bulwark role against infections: *in vitro* performances on *Candida tropicalis* biofilms

1. Morphological Analysis

Morphological analysis by light microscopy and uptake process by immunofluorescence (IF) of 3 × 10^5/cm^2 SCs, incubated with 30 µg/cm^2 AmB-MP for 5 hours at 37 °C was performed in NUNC chamber slides (LabTek II, Nunc, Thermo Fisher, Rochester, NY, USA), as previously described [51]. Briefly, the chamber slides were washed twice with D-PBS (Dulbecco's Phosphate-Buffered Saline, EUROCLONE, Milan, Italy). The washing solution was eliminated, and the cells were fixed in PFA (paraformaldehyde) 4% *w/v*, pH 7.4 for 10 minutes at room temperature (r.t.). After washing with D-PBS, cells were permeabilized with TritonX-100 at 0.1% *v/v*, washed twice with D-PBS (Lonza, Verviers, Belgium), and treated with RNAase for 10 minutes (1mg / mL, Quiagen, London, UK). Before fluorescent analysis, nuclei were counterstained with 4,6-diamino-2-phenyl (DAPI) and the slides were analyzed using a BX-41 microscope (Olympus, Tokyo, Japan) equipped with a fluorescence photo camera (F-viewer, Olympus, Tokyo, Japan); images were processed with Cell F imaging software (Olympus, Tokyo, Japan).
Bright field and fluorescence microscope observations of the uptake process showed an unchanged SCs epithelial morphology (Figure S1, panel A) and, exploiting AmB self-fluorescence, the presence of AmB-MP near the DAPI labeled nuclei of SCs was clearly detected (Figure S1, panel B).

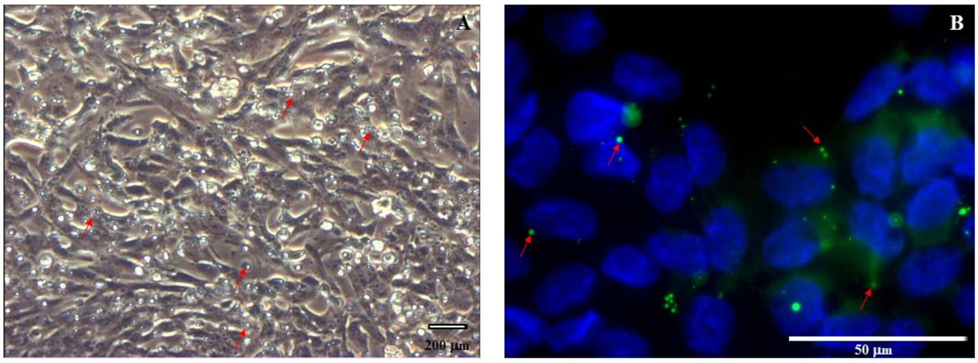

Figure S1. Morphological characterization. Light (A) and fluorescence microscope (B) of 3 × 10^5/cm^2 SCs, incubated with 30 µg/cm^2 AmB-MP for 5 hours at 37 °C. The scale bar corresponds to 200 µm for panels A and 50µm for panel B. Nuclei are counterstained with 4′,6-diamidino-2-phenylindole (DAPI) (blue) in panel B. Red arrows are representative of some AmB-MP. The images are representative of three separate experiments.

2. Biofilm inhibition test/fungal susceptibility test

To set up the best experimental conditions to test the inhibitory effect of SCs on *Candida tropicalis (C. tropicalis)* biofilm growth, different pilot experiments were performed.

To evaluate the inhibitory effect of soluble factors released by SCs, 3 × 10^5/cm^2 SC were seeded on Polyethylene Terephthalate (PET) insert to avoid direct contact with the biofilm, according to the experimental scheme described in Table S1. After the incubation times of 24 and 48 hours, the inserts were removed, SCs monolayer was observed with Optical Microscope (Bx41, Nikon, Tokyo, Japan) and the OD_{405} was read with an absorbance microplate reader (TECAN, Infinite 200 pro series, AG, Switzerland) to test the biofilm's growth.

To test the combined effect of both soluble factors and possible endocytosis by SCs, 3 × 10^5/cm^2, alone or previously loaded with blank MP and AmB-MP at the concentration of 30 μg/cm^2 were plated directly upon the biofilm of *C. tropicalis*, according to the experimental scheme in Table S1.

After the incubation times of 24 and 48 hours, plates released from the SCs monolayer were analyzed and the OD_{405} was read to test the biofilm growth state.

Ultimately, the effect of the medium conditioned for 7 days by the same number of SCs was evaluated at the same time points (Table S1, Figure S2).

The experiment showed that the growth of biofilm did not undergo substantial changes compared to *C. tropicalis* alone in the experimental set-up of *C. tropicalis* plus media conditioned by SCs or SCs placed in the insert while demonstrating that the best experimental condition was that of SCs in direct contact loaded with both blank MP and even more with AmB-MP.

Table S1. Experimental scheme of *C. tropicalis* biofilm inhibition test

**GROUPS**	**TREATMENT**
1	*C. tropicalis*
	No contact
2	*C. tropicalis + SCs on insert*
3	*C. tropicalis + SC-conditioned medium*
	Direct Contact
4	*SCs + C. tropicalis*
5	*SCs + AmB-MP + C. tropicalis*
6	*SCs + MP + C. tropicalis*

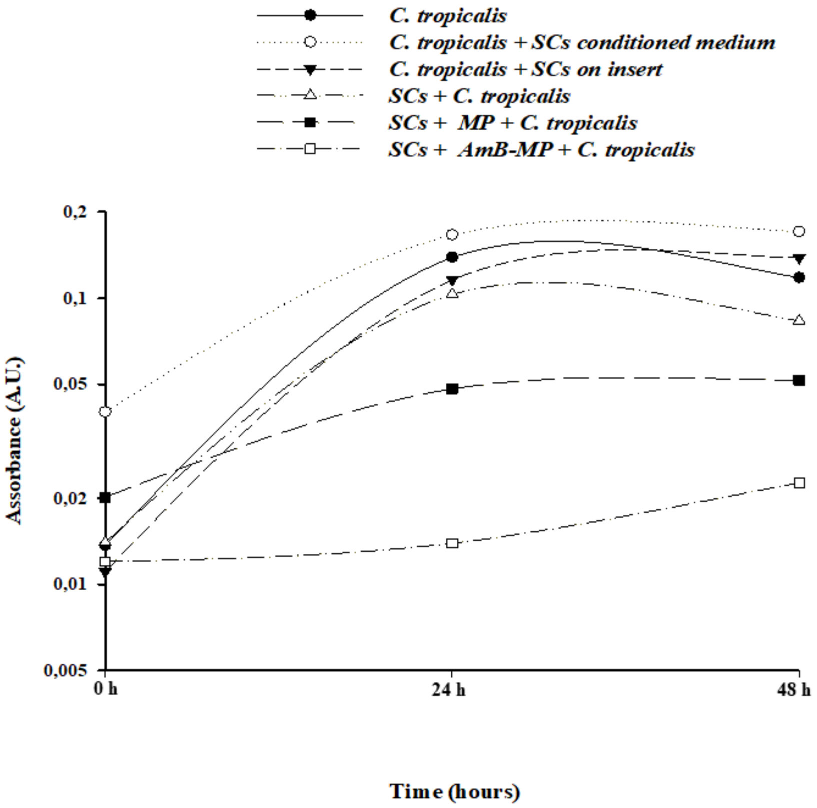

Figure S2. Biofilm inhibition test. The inhibition of *C. tropicalis* was evaluated by following the growth of the fungal biofilm for 48 hours, in the different culture conditions. *C.tropicalis*, no contact: *C. tropicalis* + SCs on insert, *C. tropicalis* + conditioned medium; direct contact: SCs + *C. tropicalis*, SCs + AmB-MP + *C. tropicalis*, SCs + MP + *C. tropicalis*. SCs concentration = 3 × 10^5/cm^2.

3. Transmission electron microscopy analysis

To study the interaction between SCs and *C. tropicalis,* samples obtained from the SCs direct co-culture condition with *C. tropicalis* for 5 hours and at the concentration of 3 × 10^5/cm^2 were fixed with 2% glutaraldehyde in 0.1 M cacodylate with 3 mM $CaCl_2$ at 4 °C overnight, and then fixed with 1% OsO_4 for 60 min. Samples were dehydrated through an ethanol series and transferred into Epon resin. One-micron-thick sections were stained with toluidine blue [77]. Ultrathin sections were cut with a diamond knife on a Reichert ultra-microtome; the sections were mounted on Formvar single-hole grids or 150-mesh grids, stained with uranyl acetate and lead citrate, and then examined at 80 kV under the electron microscope [78], (JEOL JEM 1010) (JEOLUSA, Peabody, MA, USA).

The TEM examination revealed that following 5 hours post-infection with *C. tropicalis*, SCs were viable and able to engulf the fungal spores. Moreover, they were not structurally altered, as demonstrated by the presence of perfectly intact organelles, such as a RER particularly developed and numerous mitochondria (Figure S3, panels A–E).

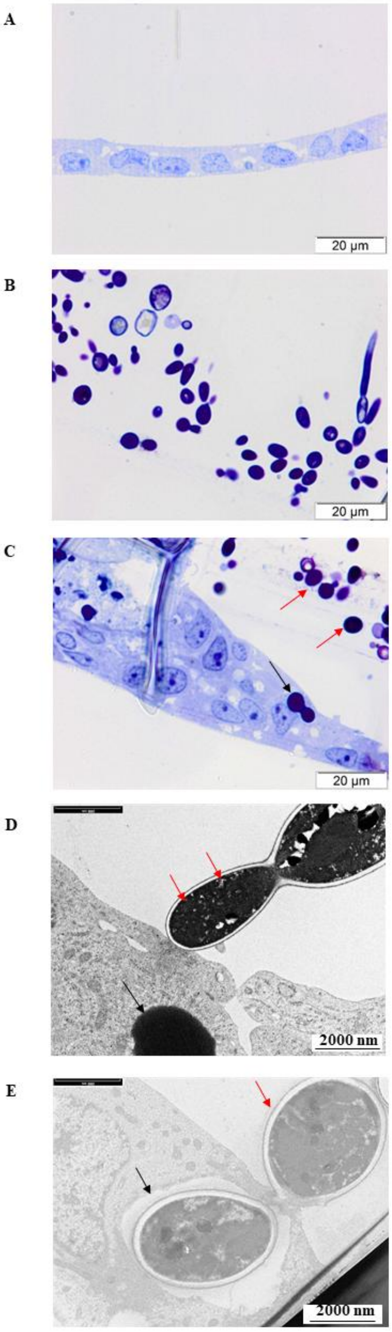

Figure S3. Representative TEM images of SCs cultures alone and in presence of *C. tropicalis.* One-micron-thick (semithin) sections were stained with toluidine blue, magnification 100×: (A) SCs monolayer (B) *C. tropicalis* pseudohyphae (C) Fungal pseudohyphae surrounding SCs (red arrows) and endocytosed by SCs (black arrow). Ultrathin sections were stained with uranyl acetate and lead citrate and examined with an electron microscope: (D) and (E) Representative images of SCs, containing quite large nuclei (N) and abundant mitochondria (M), surrounded by fungal pseudohyphae (red arrow) and endocytosed fungal pseudohyphae (black arrow).

4. Metabolic inhibiton of C. tropicalis

Through the colorimetric assay with Resazurine, the activity status/cell metabolic inhibition was evaluated within the biofilm of *C. tropicalis*. Briefly, 1 mL of Resazurine (Merck KGaA, Darmstadt, Germany) was added

in aqueous solution for well and the plates were incubated for 1 hour at 37 ° C. Following the staining, cellular metabolic status was detected by observing the plates directly: blue color indicated a metabolic quiescent state while a tone to the pink indicated that there was cellular activity [79,80].

Resazurin staining showed that biofilm development was inhibited in all the tested conditions (blue wells) and no fungal growth was observed after SCs co-culture (Figure S4, panels A-C).

These results further confirm the effective role of SCs against bacterial infections in the testis, providing hints on their potential therapeutic use in treating infectious diseases by exploiting their innate antibacterial activity and proven natural capacity to internalize drug-loaded MP [26].

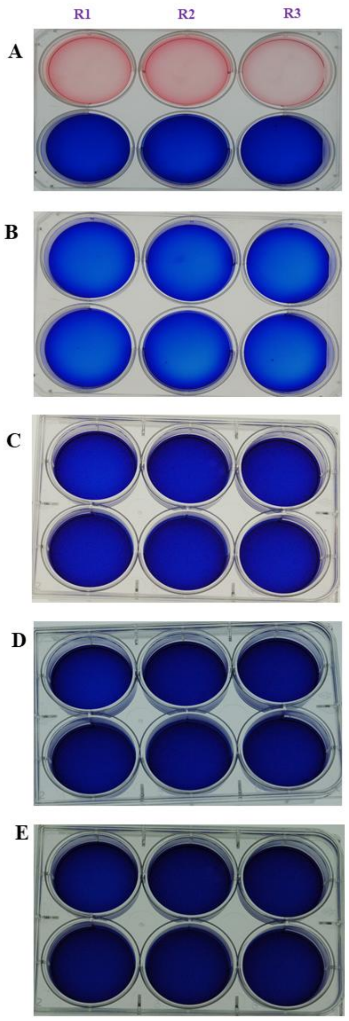

Figure S4. Metabolic inhibition of *C. tropicalis.* Resazurin staining showed that biofilm development was inhibited in all the tested conditions (blue wells indicated a metabolic quiescent state) and no fungal growth was observed after SCs co-culture (A-C) respect to *C. tropicalis* alone (pink wells indicated a cellular activity), upper panel A. Legend: R1 (replicate 1), R2 (replicate 2), R3 (replicate 3); SCs ($1 \times 10^5/cm^2$) + *C. tropicalis* (lower panel A); SCs ($1 \times 10^5/cm^2$) + MP+ *C. tropicalis* (upper panel B); SCs ($1 \times 10^5/cm^2$) + AmB-MP+ *C. tropicalis* (lower panel B); SCs ($2 \times 10^5/cm^2$) + *C. tropicalis* (upper panel C); SCs ($2 \times 10^5/cm^2$) + MP+ *C. tropicalis* (lower panel C); SCs ($2 \times 10^5/cm^2$) + AmB-MP+ *C. tropicalis* (upper panel D); SCs ($3 \times 10^5/cm^2$) + *C. tropicalis*

(lower panel D); SCs (3 × 10^5/cm^2) + MP+ *C. tropicalis* (upper panel E); SCs (2 × 10^5/cm^2) + AmB-MP+ *C. tropicalis* (lower panel E).

5. β–defensin 123 (BDF 123) secretion in culture medium

At each experimental time point, the recovered media underwent protein concentration and salt removal by spinning filtration, following Manifacturer instruction (Vivaspin, Sartorius, Göttingen, Germany). Briefly, cell culture media were collected and centrifuged (300× *g*) for 10 min to spin down any cell debris. To be sure to remove any cell debris, the supernatant was carefully collected and transferred to new 50 mL tubes and centrifuged again at 2000× *g* for 20 min.

To concentrate protein and remove salts, the obtained supernatant underwent further centrifugation with Vivaspin 3000 Da at 4000× *g* for 30 minutes. The obtained proteins, upon washing, in a volume around 100 μl of RIPA buffer, underwent Western Blotting analyses as previously reported in the Main text.

The WB analysis showed that one of the probable factors responsible for the antifungal function of SCs was BDF123, whose secretion in the culture media was detectable at 48 hours up to 96 hours, in particular in the condition of SCs + *C. tropicalis*, SCs + AmB-MP + *C. tropicalis*, SCs + MP + *C. tropicalis* (Figure S5).

48 hours

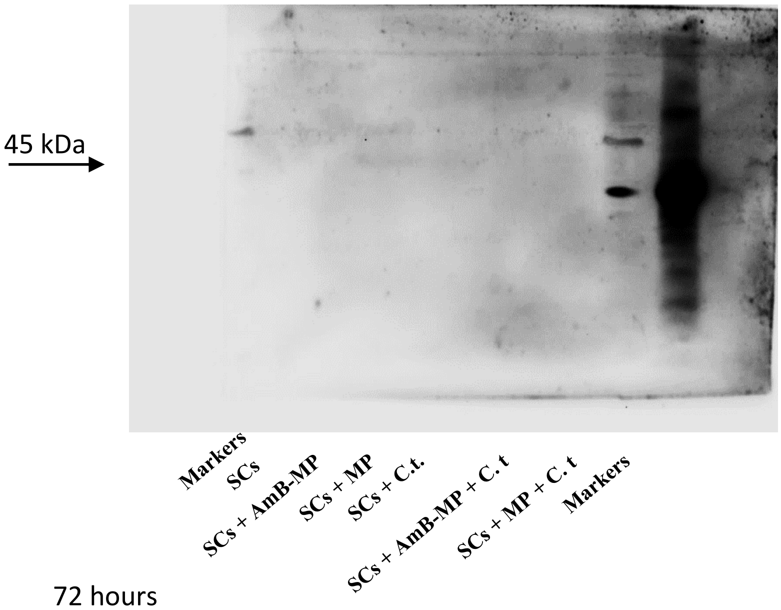

72 hours

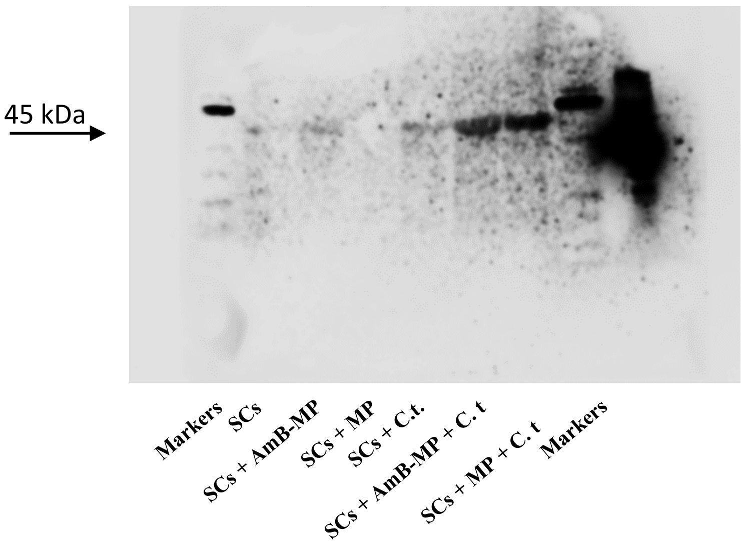

96 hours

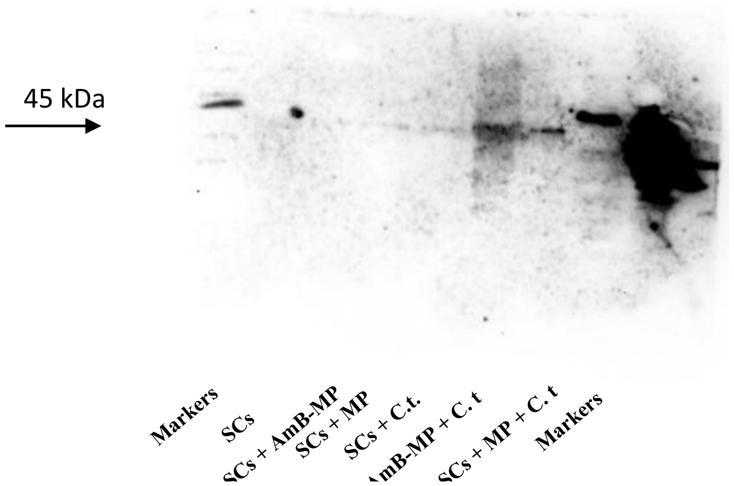

Figure S5. BDF123 secretion. The secretion of BDF123 was evaluated in the different culture conditions (A: 48 hours, B: 72 hours, C: 96 hours), after protein concentration and salt removal. SCs (1 × $10^5/cm^2$); SCs (1 × $10^5/cm^2$) + AmB-MP; SCs (1 × $10^5/cm^2$) + MP; SCs (1 × $10^5/cm^2$) + *C. tropicalis*; SCs (1 × $10^5/cm^2$) + AmB-MP+ *C. tropicalis*; SCs (1 × $10^5/cm^2$) + MP+ *C. tropicalis*.

References

26. Nagaosa, K.; Nakashima, C.; Kishimoto, A.; Nakanishi, Y. Immune response to bacteria in seminiferous epithelium. Reproduc-tion 2009, 137, 879–888. https://doi.org/10.1530/REP-08-0460.

51. Mancuso, F.; Arato, I.; Lilli, C.; Bellucci, C.; Bodo, M.; Calvitti, M.; Aglietti, M.C.; dell'Omo, M.; Nastruzzi, C.; Calafiore, R.; et al. Acute effects of lead on porcine neonatal Sertoli cells In Vitro. Toxicol Vitr. 2018, 48, 45–52. https://doi.org/10.1016/j.tiv.2017.12.013.

77. Dykstra, M.J.; Reuss, L.E. Techniques, In: Biological Electron Microscopy. Springer, Boston, MA 2003 https://doi.org/0.1007/978-1-4419-9244-4_8.

78. Ceccarelli, V.; Ronchetti, S.; Marchetti, M.C.; Calvitti, M.; Riccardi, C.; Grignani, F.; Vecchini, A. Molecular mechanisms un-derlying eicosapentaenoic acid inhibition of HDAC1 and DNMT expression and activity in carcinoma cells. Biochim Biophys Acta Gene Regul Mech 2020 1863, 194481. https://doi.org/10.1016/j.bbagrm.2020.194481.

79. Präbst, K.; Engelhardt, H.; Ringgeler, S.; Hübner, H. Basic Colorimetric Proliferation Assays: MTT, WST, and Resazurin. Methods Mol Biol 2017 1601, 1-17. https://doi.org/10.1007/978-1-4939-6960-9_1.

80. Fai, P.B.; Grant, A. A rapid resazurin bioassay for assessing the toxicity of fungicides. Chemosphere 2009, 74, 1165-1170. https://doi.org/10.1016/j.chemosphere.2008.11.078.
